# Complex Consequences of Herbivory and Interplant Cues in Three Annual Plants

**DOI:** 10.1371/journal.pone.0038105

**Published:** 2012-05-31

**Authors:** Ian S. Pearse, Lauren M. Porensky, Louie H. Yang, Maureen L. Stanton, Richard Karban, Lisa Bhattacharyya, Rosa Cox, Karin Dove, August Higgins, Corrina Kamoroff, Travis Kirk, Christopher Knight, Rebecca Koch, Corwin Parker, Hilary Rollins, Kelsey Tanner

**Affiliations:** 1 Department of Entomology, University of California Davis, Davis, California, United States of America; 2 Department of Plant Sciences, University of California Davis, Davis, California, United States of America; 3 Department of Evolution and Ecology, University of California Davis, Davis, California, United States of America; Centro de Investigación y de Estudios Avanzados, Mexico

## Abstract

Information exchange (or signaling) between plants following herbivore damage has recently been shown to affect plant responses to herbivory in relatively simple natural systems. In a large, manipulative field study using three annual plant species (*Achyrachaena mollis*, *Lupinus nanus*, and *Sinapis arvensis*), we tested whether experimental damage to a neighboring conspecific affected a plant's lifetime fitness and interactions with herbivores. By manipulating relatedness between plants, we assessed whether genetic relatedness of neighboring individuals influenced the outcome of having a damaged neighbor. Additionally, in laboratory feeding assays, we assessed whether damage to a neighboring plant specifically affected palatability to a generalist herbivore and, for *S. arvensis*, a specialist herbivore. Our study suggested a high level of contingency in the outcomes of plant signaling. For example, in the field, damaging a neighbor resulted in greater herbivory to *A. mollis*, but only when the damaged neighbor was a close relative. Similarly, in laboratory trials, the palatability of *S. arvensis* to a generalist herbivore increased after the plant was exposed to a damaged neighbor, while palatability to a specialist herbivore decreased. Across all species, damage to a neighbor resulted in decreased lifetime fitness, but only if neighbors were closely related. These results suggest that the outcomes of plant signaling within multi-species neighborhoods may be far more context-specific than has been previously shown. In particular, our study shows that herbivore interactions and signaling between plants are contingent on the genetic relationship between neighboring plants. Many factors affect the outcomes of plant signaling, and studies that clarify these factors will be necessary in order to assess the role of plant information exchange about herbivory in natural systems.

## Introduction

Plants alter their phenotypes in response to cues that provide information about their neighbors [1], [2]. One example of plant-plant interactions is plant responses to cues released by neighbors that are attacked by herbivores, and we now have at least ten well-accepted examples of plants that adjust their phenotypes in response to cues released by damaged neighbors (reviewed recently by [3]). In most of these cases, plants sense a volatile cue from a damaged neighbor and induce defensive metabolites, sensitivity to future damage, or anatomical structures in order to defend themselves from their herbivores [4], [5], [6], [7], [8]. The defensive response may be adaptive if damage to the neighbor forecasts an increase in herbivore pressure to the plant receiving the cue.

There is good reason to suspect that relationships among cue-emitting and cue-receiving plants may alter a plant's response to a damaged neighbor. An individual may be more likely to respond to the cues released by a close relative for at least three reasons: 1) kin selection may favor honest signals between related neighbors, 2) the emitter and receiver may share traits that shape resistance or susceptibility to particular herbivores, and 3) the cue may be more easily recognized, especially if cues and receptors are variable among individuals [9], [10], [11], [12]. Although plant biologists have only recently considered whether individuals perceive and respond differently to cues based on relatedness, several empirical examples involving plant competition suggest that this property may be important. Roots of different individuals of *Ambrosia dumosa* that came into contact inhibited each other to a much greater extent than roots connected to the same individual [13], [14], [15], and a similar trend has been found in the roots of several other species [16], [17], [18]. Root growth has also been found to differ in interactions between kin and unrelated conspecifics of *Cakile edentula* [19], and plant relatedness influences other growth parameters in *Impatiens pallida* [20]. Despite the potential importance of neighbor relatedness for the evolution and ecology of plant-to-plant information exchange, there is little known about whether plants respond differently to cues emitted by relatives compared to those from strangers. Two plants, *Phaseolus lunatus* and *Artemesia tridentata*, respond more to damage-induced volatile cues from the same individual or genet than from other genotypes [8], [21]. Responses of spotted knapweed individuals induced by adding jasmonic acid were different if neighbors were conspecifics versus heterospecifics [22].

We have little information on the fitness consequences of cues between neighboring plants in natural plant communities and even less information about how plant relatedness affects this interaction. Most examples of plant responses to volatile cues have documented short-term changes in growth or herbivory in long-lived woody plants, vines or agricultural crops (but see work on wild tobacco [23]). Accordingly, the lifetime fitness consequences for individuals either emitting cues or responding to them are poorly known [3], [8], [23], [24]. Furthermore, information exchange has been assessed in relatively few plant species, and since negative results are rarely reported, we have little sense about how widespread or species-specific induced responses to cues released by damaged neighbors might be (similar to the problem reported in [25]). Likewise, the consequences of damage-induced cues have never been tested in different contexts of plant relatedness, despite genetic relationships being important for other species interactions. Together, these limitations mean that we do not know whether the patterns emerging from experimental studies so far are representative of how information exchange between plants operates in complex natural communities.

In this study, we worked with three common annual plant species in a low-elevation California grassland to address several outstanding questions involving wounding-induced plant to plant cues. In a factorial experiment, we manipulated wounding to a neighbor (an “emitter” plant) as well as the relatedness between the emitter and two “receiver” plants for each of the three plant species. On the first receiver plant, which was planted in the field next to the emitter plant, we assessed the accumulation of natural herbivore damage, plant growth parameters, and lifetime plant fitness. The second receiver plant was planted in a pot, placed adjacent to the emitter plant, and used for a laboratory palatability assay with either a generalist or specialist herbivore. With this design, we were able to assess 1) the impacts of wounding-induced plant cues on subsequent herbivore damage and lifetime plant fitness, 2) the interplay between plant relatedness and wounding-induced cues, 3) the species-specificity of wounding-induced cues, and 4) the consistency between palatability bioassays and field herbivory as responses to wounding-induced cues.

## Methods

### Study system and species

We performed our field work from February to May 2011 in the Ecology Lab grassland site on the University of California, Davis campus (+38°31′47.24″, −121°46′53.58″). The site experiences a Mediterranean climate, with rainy winter months and a long summer drought. As is typical of low-elevation California annual grassland habitat, vegetation at the site is comprised mostly of non-native species of Mediterranean origin, though some native annuals are present.

To test for the generality of plant signaling across different plant taxa, we performed our experiment on three species which had not been previously studied in this context and which were from different plant families. We selected three species for our trials: *Lupinus nanus* (Fabaceae), *Achyrachaena mollis* (Asteraceae), and *Sinapis arvensis* (Brassicaceae). Seeds of all three species were collected within the past ten years as maternal seed families from field sites within 100 km of our study site. *A. mollis* grows naturally and in abundance at our field site. Both *L. nanus* and *S. arvensis* are common annuals in Californian grasslands in the area, but are not currently present at the site. *L. nanus* and *A. mollis* are both California natives, and *S. arvensis* is a naturalized, weedy plant of European origin [26].

For laboratory bioassays of plant palatability, we used lepidopteran neonates. We procured *Spodoptera exigua* (a generalist feeder) from Marrone Bio Innovations (Davis, CA) and *Pieris rapae* (a specialist on Brassicaceae) from Carolina Biological Supply Company (Burlington, NC).

### Experimental set-up and design

At the study site, we laid out 180 plots in a regular 12×15 plot grid. Plots measured ∼60×60 cm and were separated by 2 m center-to-center. We removed all above-ground vegetation from each plot during initial set-up and continued to remove weeds as they emerged until our experimental wounding treatments began. Each plot was covered with black Dewitt Weed Barrier Pro into which we cut 3 circular holes. The three holes were spaced 7.5 cm apart. We planted a designated ‘emitter’ in the central hole and a ‘field receiver’ in the northern hole. The third (southern) hole was assigned to potted ‘bioassay receivers’ to be used in palatability assays. As emitters and field receivers grew in the field for the duration of the experiment, their canopies and probably their rooting zones began to overlap. Bioassay receivers were grown under greenhouse conditions, briefly placed in the field within their pots and then returned to the laboratory for palatability testing, preventing root interactions between the potted bioassay receivers and emitters. The small space between neighboring plants allowed leaves to overlap and maximized the potential interaction of airborne cues between plants.

The 180 field plots were randomly assigned to different experimental treatment combinations. Treatments comprised a 2×2×3 factorial design in which we varied neighbor relatedness (emitter and receiver were either “related” or “unrelated”) and emitter-wounding (“wounded” or “unwounded”) for each of the three plant species. In the related-neighbor treatment, neighbors were from the same field-collected maternal family, whereas in the unrelated treatment, receivers were from a known maternal family and emitters were grown from bulk-collected seeds of unknown maternity. In plots assigned to the wounded-neighbor treatment, the emitter was mechanically damaged during an experimental test period. No plants were experimentally damaged in unwounded-neighbor plots. We aimed to have 15 replicates (one per maternal plant family) of each species*relatedness*wounding treatment combination. Fatalities and incomplete seedling emergence led to uneven replication across treatments and families.

### Seedling preparation and planting

To generate experimental emitters and field receivers, we planted seeds into plug flats roughly 2–3 weeks before transplanting seedlings into the field site. Germination occurred on a greenhouse mist bench, and then flats of seedlings were placed into a lath-house to harden for several days before planting into the experimental plots. Four weeks later, we planted bioassay receivers as single seeds into 66 mL “Conetainer” elongated pots (Steuwe and Sons; Tangent, OR). These pots exclude root contact between bioassay receivers and other plants. All seeds were planted in modified UC Mix planting medium (UC Davis, Davis, CA). Before planting, *L. nanus* seed coats were scarified with a razor blade and *A. mollis* pappi were removed. We maintained flats under natural day-length conditions on a mist bench in UC Davis's Orchard Park Greenhouse during germination and early growth.

We planted *S. arvensis* and *L. nanus* seedlings into the field on 7 March, then planted the slower-growing *A. mollis* seedlings on 14 March. Transplant fatalities were replaced within 10 days of initial planting; subsequent fatalities were not replaced. After planting, we watered each seedling with approximately 250 mL of water 2–3 times a week except during substantial rain. We applied fertilizer (Miracle Grow: Nursery Select Professional Formula - All purpose Plant Food; concentration of 0.5 g/L) approximately every two weeks throughout the establishment phase of the experiment. From planting (7 March) until two weeks prior to the initiation of wounding treatments (11 April), we applied approximately 0.4 oz of molluscicide around or within each plot about every two weeks (Ortho: Bug-Geta - Snail and Slug Killer; Active ingredient 3.25% Metaldehyde).

### Experimental wounding and data collection

Experimental wounding treatments were imposed on 25 April and 2 May 2011. On 25 April, we mechanically damaged 50% of each emitter's leaf area in plots assigned to the ‘wounded-neighbor’ treatment using a florist's pin “frog” for *S. arvensis* or pliers for *A. mollis* and *L. nanus*. On 2 May, the remaining leaf area on each emitter was damaged. Before initiating leaf damage on each wounding date, we placed one bioassay receiver seedling into each plot, sinking the pot into a previously prepared hole until the soil in the pot was at ground level. On 25 April we performed a baseline assessment of each plant (emitter, field receiver and bioassay receiver) within each plot. To obtain an approximate measure of plant size, we measured the number of leaves and the length (mm) of the longest leaf of all plants. We then visually assessed two types of herbivore damage – leaf removal and other kinds of damage (e.g. spotting, desiccation) – using a scale of 0–100 percent of total leaf area. Additionally, we recorded aphid and mirid abundance on all plants. On 2 May we assessed herbivore damage and plant stage for field receivers and repeated our palatability bioassay with a new group of bioassay receivers in each plot. On 9 May and 16 May we assessed herbivore damage and plant phenological stage for all field receivers.

Two days after each wounding treatment, we transferred bioassay receivers to the lab for a palatability trial with a generalist herbivore. Immediately after transferring bioassay receivers to the lab, we performed ‘pre-trial’ damage assessments and placed one freshly-hatched *Spodoptera exigua* neonate on the largest lower leaf of each plant. To discourage *Spodoptera* from leaving their host plants, we enclosed each pot in a fluon-lined paper drinking cup. Fourteen days later, we measured the percent of leaf area eaten on each bioassay receiver. We also observed the presence of frass from *Spodoptera* caterpillars as evidence of the initiation of *Spodoptera* feeding.

To contrast the palatability of bioassay receivers to generalist versus specialist herbivores, we also initiated a feeding trial with *Pieris rapae*, a specialized herbivore of *S. arvensis*. *Pieris* neonates were placed on *S. arvensis* plants on 4 May and were allowed to feed for 7 days. At the end of the trial, we recorded percent leaf removal by *Pieris* and caterpillar mass.

### Statistical analyses

To investigate the impacts of neighbor-wounding and relatedness on herbivore damage to field receivers we used linear mixed models with maximum likelihood estimation and Satterthwaite's approximation for degrees of freedom. We included species, wounding treatment, relatedness treatment, survey date (2 May, 9 May or 16 May) and all possible interactions as fixed effects. We treated survey date as a nominal factor to account for the possibility that damage might vary non-linearly with date. The receiver plant's maternal family was included as a random effect. We addressed the non-independence of repeated surveys by treating consecutive surveys within the same plot as repeated measures (including observations on bioassay plants that were introduced and assayed at different time points). All data were log-transformed to meet ANOVA assumptions.

To assess treatment effects on end-of-season biomass and fruit and seed production, we ran mixed models with species, neighbor-wounding treatment, relatedness treatment and all possible interactions as fixed effects. In each model, the receiver plant's maternal family was included as a random effect.

For all four response variables, possible covariates included various measures of natural herbivore damage and herbivore counts made prior to the initiation of the experimental damage treatment, as well as plant size and plant phenological stage for both the emitter and receiver on 25 April, the date of the first experimental wounding. We selected among possible covariates by initially including all covariates in the model and then sequentially removing covariates using a backwards stepwise method with a *p*-value cutoff of 0.05.

To detect possible effects of neighbor damage and relatedness on the phenology of field receivers, we created a generalized linear model with receiver plant stage on 9 May as a binomial response variable. This model included the length of the longest leaf at the onset of the emitter damage treatments to account for pre-treatment differences in plant development. Although we recorded three categories of plant stage (rosette, bolting, flowering), it was possible to analyze as a binomial variable, because each species exhibited no more than 2 stages on 9 May. *S. arvensis* was excluded from the phenology analysis, as it exhibited no variation in plant stage on 9 May.

If any interactions involving species were marginally significant (P<0.1) in the final model, we investigated simple effects by re-running the model (with the same covariates) separately for each species. Similarly, we investigated simple effects if we detected significant interactions between relatedness and wounding treatments.

To investigate impacts of relatedness and wounding treatments on the palatability of bioassay receiver plants, we used linear mixed models as described above with log-transformed percent leaf removal (on bioassay receivers) as the response variable. We ran one model for the *Spodoptera* assay (which was performed twice on *A. mollis* and *L. nanus* and once on *S. arvensis*) and one model for the *Pieris* assay (performed once on *S. arvensis*). For the *Spodoptera* assay we included species, emitter wounding treatment, neighbor relatedness and all possible interactions as fixed effects, and maternal family as a random factor. We also included treatment date (damage on either 25 April or 2 May) as a random factor, and we treated consecutive bioassay receiver measurements from the same field plot as repeated measures. For the *Pieris* assay we included emitter wounding treatment, neighbor relatedness and their interaction as fixed effects, and maternal family as a random factor. For both assays, covariate selection and treatment of interactions followed procedures for the field experiment.

The initiation of *Spodoptera* or *Pieris* feeding behavior was identified by the presence of frass on the bioassay receiver. To assess effects of neighbor treatments on feeding behavior, we constructed a generalized linear model with *Pieris* or *Spodoptera* feeding as a binomial response variable. None of the covariates reached significance in the full model, and so all were removed. As the second *Spodoptera* feeding trial contained few individuals from each treatment, convergence in binomial repeated-measures models was never reached, so only the first feeding trial was analyzed for all binomial responses.

All analyses on continuous response variables were conducted in SAS 9.2 (SAS Institute Inc., Cary, North Carolina USA) using the MIXED procedure. Analyses on nominal response variables (phenology and frass presence) were conducted in R [27] using package *car* [28]. Data from this study are made available at Dryad (doi:10.5061/dryad.f1c5j).

## Results

### Field receivers—treatment effects on damage, fitness and phenology

**Figure 1:**
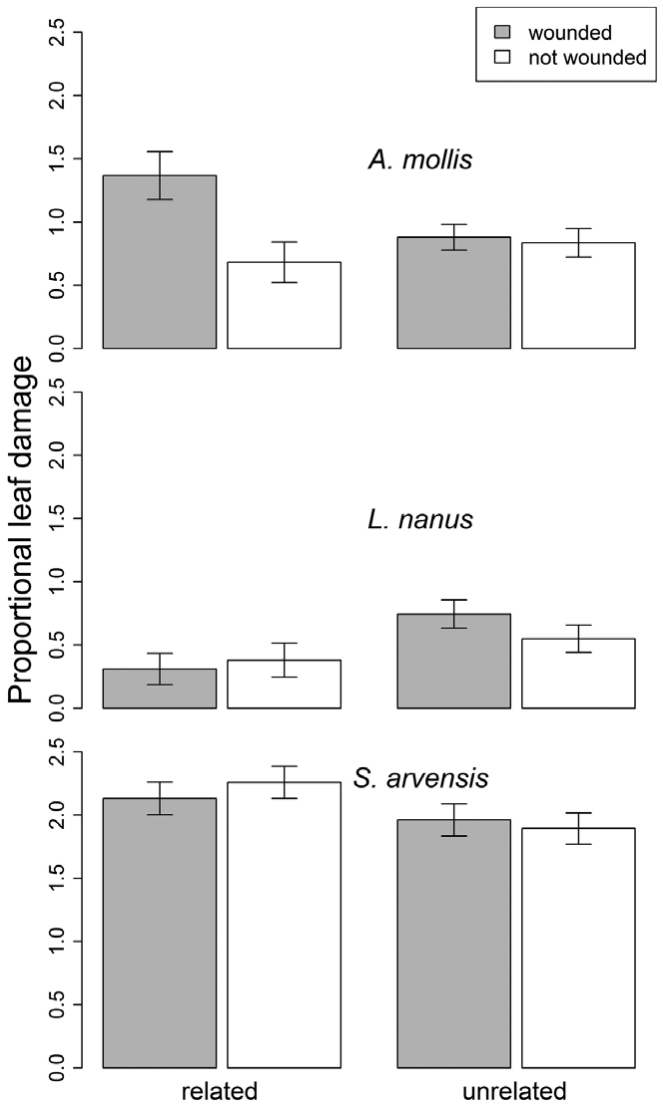
The effect of neighbor wounding and relatedness (maternal siblings or unrelated) on subsequent herbivore damage (log-transformed percent leaf damage) to conspecific neighbors of three experimental plant species in the field. Least Square Mean +/− SE.

Across the whole experiment, field receivers growing next to experimentally wounded neighbors received significantly more damage than those with unwounded neighbors, but this overall effect depended on relatedness and was largely due to the responses of *A. mollis* (Table 1). When *A. mollis* emitters and receivers were related, experimentally wounding the emitters increased average leaf removal on field receivers by 55%, and this increase was significant on some survey dates but not others (Fig. 1a, Table S1, simple effect of wounding*date: df = 2,35; F = 5.01, P = 0.01). However, experimental wounding did not substantially increase leaf removal when neighbors were unrelated (Fig. 1a, Table S1, simple effect P-values>0.2). In contrast, damage to the emitter had no significant effect on the damage experienced by *L. nanus* (Fig. 1b) or *S. arvensis* (Fig. 1c) receivers, regardless of whether they were related to their neighboring emitter (Table S1). For these latter two species, we detected main effects of neighbor relatedness on damage to field receivers that were largely independent of the emitter damage treatments. In *L. nanus*, related receivers experienced 47% less leaf removal than unrelated receivers (Fig. 1b, Table S1). *S. arvensis* receivers displayed a trend opposite to that of *L. nanus*; related receivers experienced 12% more leaf removal than unrelated receivers overall, and this trend was most pronounced after the second application of neighbor wounding (Fig. 1c, Table S1, simple effect of neighbor relatedness for second survey date: df = 1,57; F = 5.38, P = 0.02).

**Table 1:** Mixed model results for effects of a neighbor damage treatment, plant species and neighbor relatedness treatments on leaf damage to “receivers” in the field.

Effect	num DF	den DF	F Value	Pr>F	estimate	std err
**species**	**2**	**145**	**12.23**	**<.0001**		
**wounded**	**1**	**140**	**4.02**	**0.05**		
species*wounded	2	140	1.60	0.21		
neighbor relatedness	1	140	0.05	0.82		
species*neighbor relatedness	**2**	**141**	**4.28**	**0.02**		
wounded*neighbor relatedness	1	141	0.10	0.76		
species*wounded*neighbor relatedness	2	141	2.13	0.12		
**date**	**2**	**142**	**22.16**	**<.0001**		
species*date	**4**	**142**	**9.26**	**<.0001**		
wounded*date	2	142	1.35	0.26		
species*wounded*date	4	142	1.88	0.12		
neighbor relatedness*date	2	142	0.36	0.70		
species*neighbor relatedness*date	4	142	0.37	0.83		
wounded*neighbor relatedness*date	**2**	**142**	**4.27**	**0.02**		
species*wounded*neighbor relatedness*date	4	142	0.21	0.94		
**pre-treatment leaf damage (receiver)**	**1**	**143**	**35.51**	**<.0001**	0.025	0.004
**leaf count (receiver)**	**1**	**149**	**9.32**	**0.00**	−0.008	0.003
**leaf length (receiver) (emitter)**	**1**	**143**	**7.72**	**0.01**	0.005	0.002
**pretreatment mirid abundance (receiver)**	**1**	**142**	**9.89**	**0.00**	0.298	0.095
**pretreatment aphid abundance (emitter)**	**1**	**142**	**4.54**	**0.03**	−0.285	0.134

Models account for sampling date and covariate factors relating to plant size and pre-treatment damage levels. Emphasis indicates significance at the P = 0.05 level.

**Figure 2:**
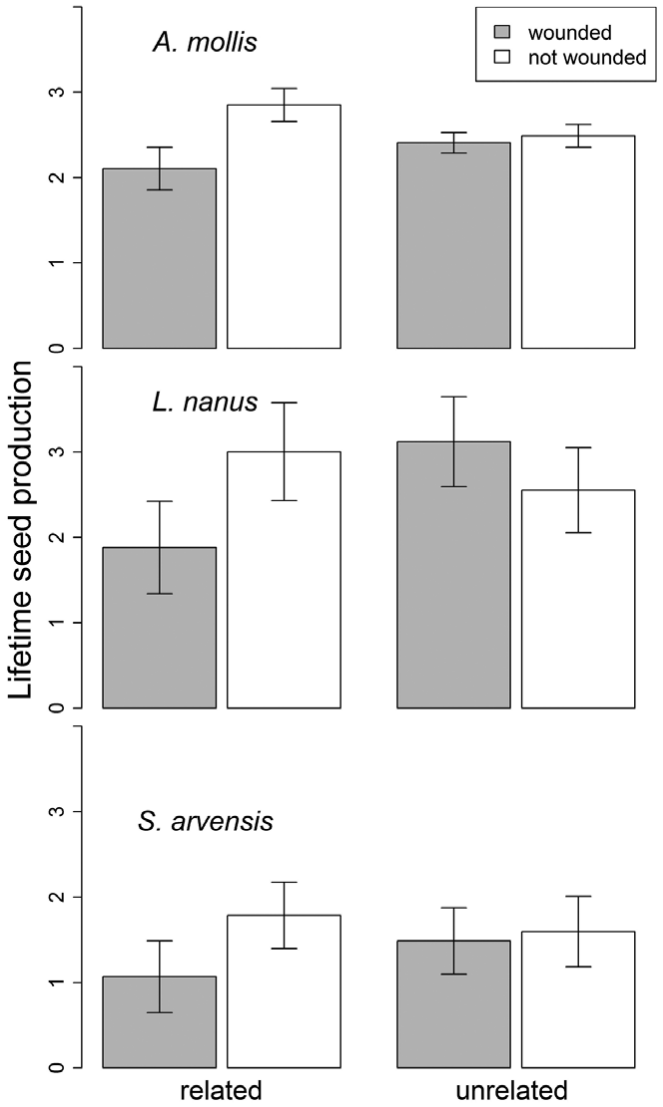
The effect of neighbor wounding and relatedness (maternal siblings or unrelated) on lifetime seed production (log-transformed) of three experimental plant species in the field. Least Square Mean +/− SE.

Across all three species, the effect of neighbor wounding on receiver fitness indicators depended significantly upon whether or not the neighbors were related (Tables S2, S3, S4). For related receivers, neighbor wounding had a marginally significant effect on plant fitness, reducing seed production and fruit production by an average of 30% and 31% respectively (Figs. 2 and S2, Table S3, Table S4, simple effect of wounding for seeds: df = 1,60; F = 3.2; P = 0.08; pods: df = 1,60; F = 2.68; P = 0.11). For unrelated receivers, neighbor wounding had no significant effect on fruit or seed production (Figs. 2 and S2, Table S3, simple effect P-values>0.2). Similarly, neighbor wounding marginally reduced final receiver biomass by 20% for related receivers (simple effect df = 1,50; F = 2.9; P = 0.09, Fig. S1), while for unrelated receivers, neighbor wounding actually increased final receiver biomass by 16% (simple effect df = 1,87; F = 4.81; P = 0.03, Fig. S1).

**Figure 3:**
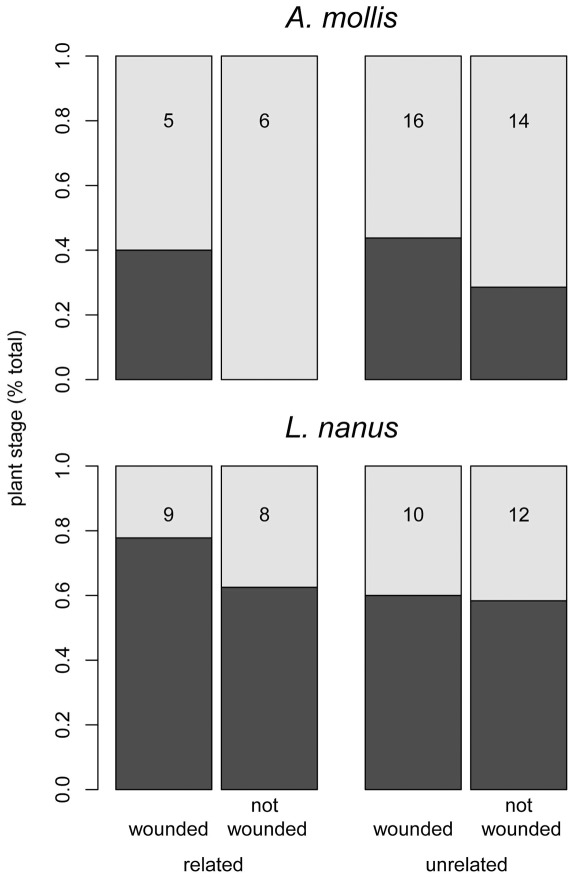
The effect of neighbor wounding and relatedness (maternal siblings or unrelated) on developmental phenology of conspecific neighbors of three experimental plant species in the field. Filled bars are the number of bolting plants, and empty bars are the number of flowering plants in each treatment two weeks after the wounding of neighboring plants (May 9, 2011). The total number of plants in each treatment is shown on each bar.

Wounding of neighbors affected the phenology of field receivers in some plant species but not others (Table S5). Specifically, *A. mollis* individuals experienced a delayed developmental phenology when their neighbors were wounded (Fig. 3, Table S6). Wounding of neighbors did not affect the development of *L. nanus* (Fig. 3), and *S. arvensis* individuals exhibited no variation in developmental stage during the observation period.

### Palatability bioassays with bioassay receivers

**Figure 4:**
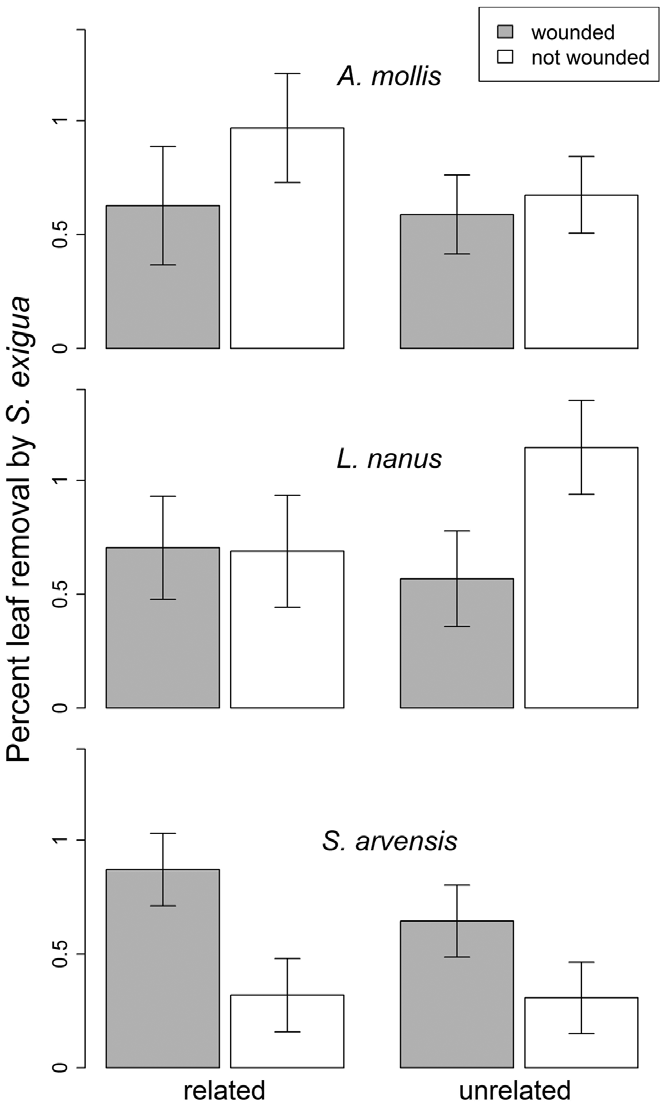
The effect of neighbor wounding and relatedness (maternal siblings or unrelated) on the percent leaf area (log-transformed) of conspecific neighbors of three potted experimental plant species consumed by generalist *Spodoptera* caterpillars (as an indicator of leaf palatability to generalists). The potted plant was exposed to a damaged or undamaged neighbor in the field for 2 days. At this point the plant was moved indoors, and a feeding trial with a neonate *Spodoptera* caterpillar was initiated. Least Square Mean +/− SE.

Impacts of experimental neighbor-wounding on generalist herbivore damage to bioassay receiver plants appeared to vary among species (Table S7). In *A. mollis* and *L. nanus*, wounding and relatedness had no significant impacts on the amount of leaf material eaten by generalist *Spodoptera* larvae (Fig. 4, Table S8). In *S. arvensis*, however, emitter wounding increased leaf removal on receivers by 141% (Fig. 4, Table S8). Likewise, 8 times more *Spodoptera* individuals initiated feeding on *S. arvensis* plants with wounded neighbors than fed on plants with unwounded neighbors, but the wounding of a *L. nanus* or *A. mollis* neighbor had no effect on *Spodoptera* feeding initiation (Fig. S3, Table S9, S10). Palatability of bioassay receivers to generalist *Spodoptera* caterpillars was not influenced by neighbor relatedness in any of the three species.

**Figure 5:**
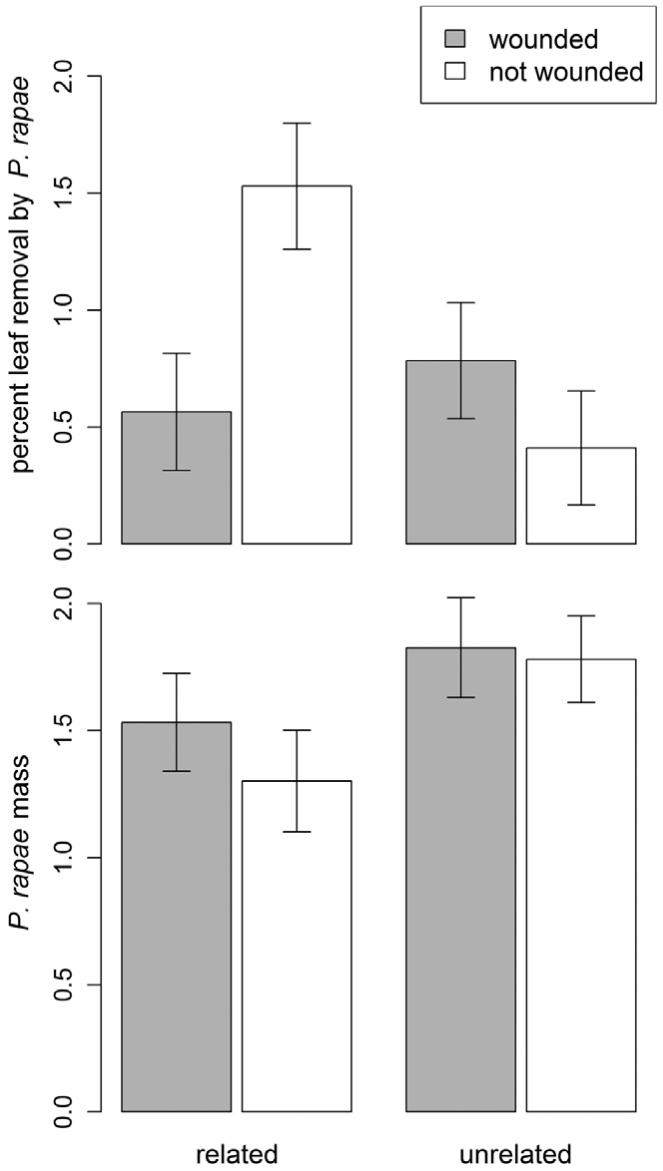
For the crucifer *Sinapis arvensis*, the effect of neighbor-wounding and relatedness (maternal siblings or unrelated) on various measures of leaf palatability to a specialist herbivore (*Pieris*). The potted plant was exposed to a damaged or undamaged neighbor in the field for 2 days. At this point the plant was moved indoors, and a no-choice feeding trial with a neonate *Pieris* caterpillar was initiated. Graphs show a) the percent leaf area removed by *Pieris* (log-transformed), and b) *Pieris* caterpillar mass (log-transformed) at the end of the feeding trial. Bars indicate least square mean +/− SE.

Specialist *Pieris* caterpillars responded very differently to experimental treatments on *S. arvensis* than did generalist *Spodoptera*. When *S. arvensis* emitters and receivers were related, wounding the emitter reduced *Pieris* damage to the bioassay receiver (Fig. 5a, Table S11; simple effect df = 1,14; F = 18.15; P = 0.0008). For unrelated neighbors, neighbor-wounding increased *Pieris* damage (Fig. 5a, Table S11; simple effect df = 1,17; F = 11.9; P = 0.003). *Pieris* caterpillars gained significantly more weight when receivers were unrelated to neighboring emitters, regardless of wounding treatment (Fig. 5b, Table S12).

## Discussion

Our study was designed to assess the consequences of plant-to-plant information exchange for herbivory rates and plant fitness in a realistic plant community. The results from our field study contrast sharply with previous experiments in which damage to neighboring plants consistently led to reduced susceptibility to herbivores [3], [6], [24] and greater plant fitness [8], [23]. Instead, we found that the effects of damage to a neighbor in the field depended on the plant species and the relatedness of the neighbor. In striking contrast to previous studies, damage to neighbors (emitter plants) decreased various measures of receiver fitness (Fig. 2) in all three plant species in the field, and in *A. mollis*, receivers experienced more damage (Fig. 1) and delayed phenology (Fig. 4) when neighbors were experimentally damaged.

When significant treatment effects were observed in laboratory feeding trials with a generalist herbivore, they tended to parallel patterns observed in the field. The similarity between the palatability assays and the field damage observations persisted despite differences in root contact and duration of exposure to emitter volatiles between field receiver plants versus bioassay receiver plants. For example, in *S. arvensis*, damage to the emitter plant increased the leaf tissue that *Spodoptera* caterpillars consumed on bioassay receivers that were placed in the field for only two days (Fig. 4) and increased the likelihood that *Spodoptera* caterpillars would initiate feeding (Figure S3). The only case in which damage to an emitter plant resulted in evidence for induced resistance in a receiver plant was in laboratory feeding trials in which *S. arvensis* receivers were challenged by a specialist herbivore. In this case, damage to a related emitter plant decreased the leaf tissue that *Pieris* caterpillars consumed, but had no effect on *Pieris* weight gain (Fig. 5).

**Figure 6:**
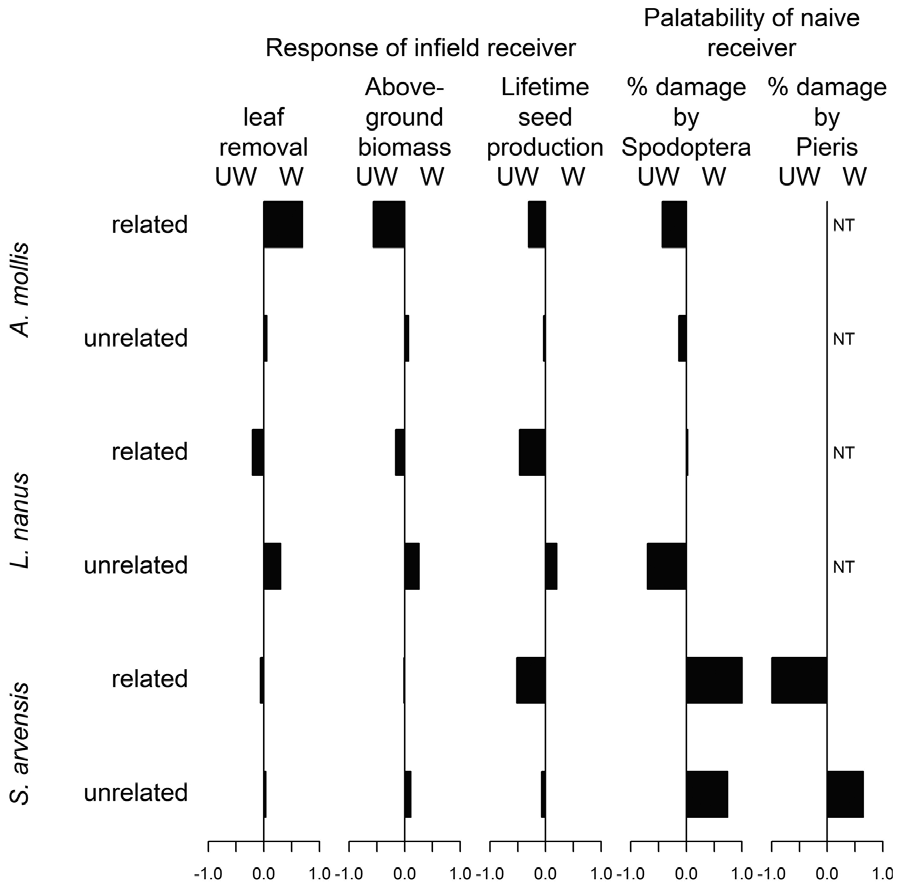
A summary of experimental results. For each response variable, the bar represents the log response ratio where the conspecific neighbor (emitter) was experimentally wounded (W) versus unwounded (UW). A positive bar indicates that the response variable of the receiver plant was greater when the emitting plant was wounded than when it was unwounded; a negative bar indicates the converse. The effect of neighbor wounding is shown both related and unrelated pairs of each of the three plant species in this study. For example, in related pairs of *A. mollis*, wounding to a neighbor increased the leaf removal that a focal plant suffered compared to focal plants with unwounded neighbors. Palatability to a specialist herbivore (*Pieris*) could only be assessed for one plant species (*S. arvensis*) and was not tested (NT) for other plant species.

The effects of damage-induced plant cues on neighboring plants were often highly dependent on whether the emitter and receiver plants were genetically related to one another (Fig. 6). For example, damage to a neighbor resulted in higher natural levels of herbivory to *A. mollis*, but this trend was only apparent when neighboring plants were close relatives (Fig. 1). Likewise, in all three plant species, damage to a neighbor reduced the receiver's lifetime seed production (and other fitness measures) only when that neighbor was a close relative (Figs. 2, S1, S2). Finally, in lab feeding trials with the specialist herbivore *Pieris*, damage to a neighboring plant decreased *Pieris* feeding only when the neighbor was related to the focal plant (Fig. 5). As these examples suggest, we found that the consequences of having a wounded neighbor were generally stronger when the neighboring plant was a close relative (Fig. 6). Our results strongly suggest that the genetic relationships among neighbors within a plant population are an important component of plant-herbivore interactions [29], [30], and that genetic relatedness influences the transfer of information between plants [19], [21].

**Figure 7:**
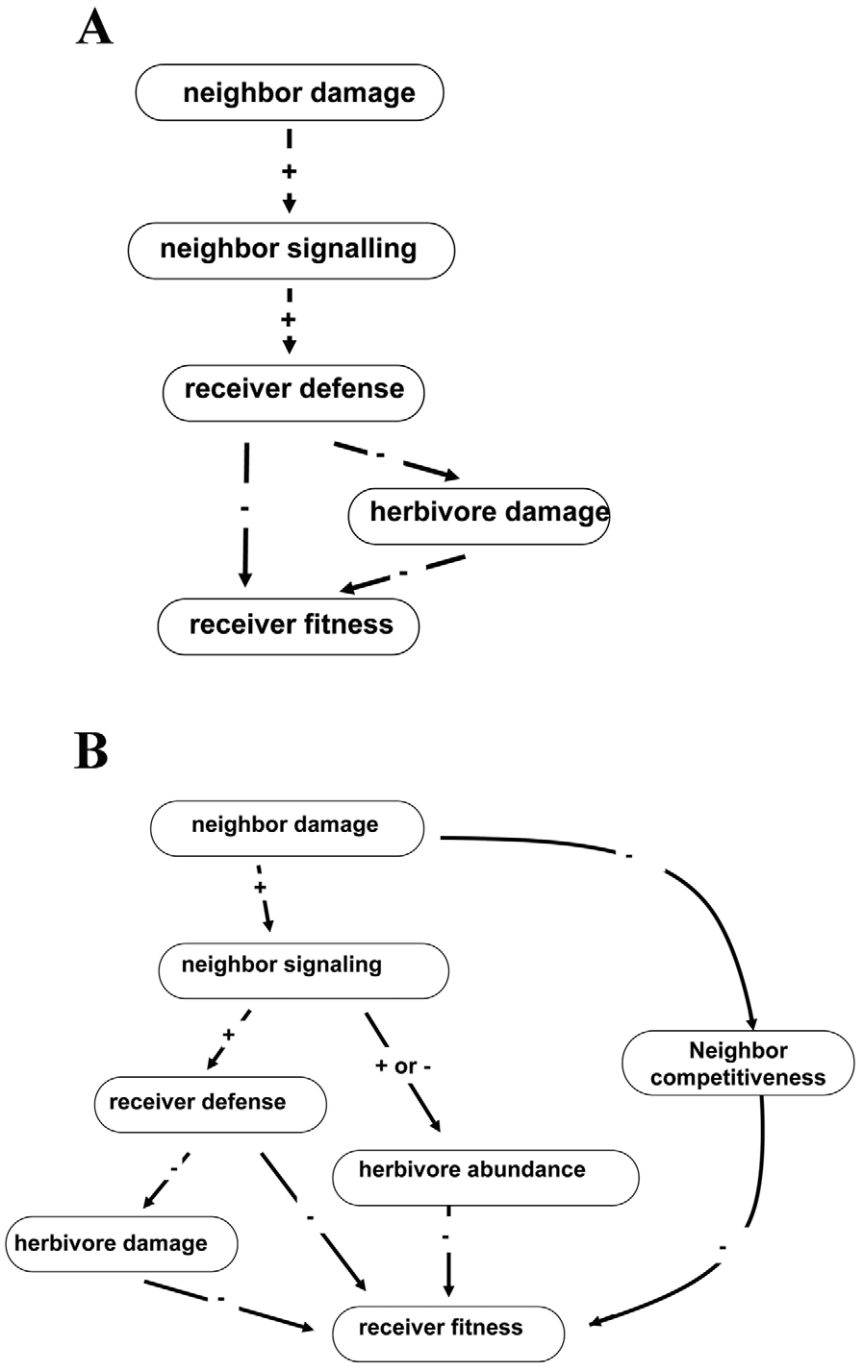
A conceptual diagram of potential ecological interactions. a) Interactions predicted by plant signaling theory, and b) a more generalized scheme showing possible outcomes of damage to neighboring plants in a complex community setting.

The complex, often indirect ecological interactions that occur in natural settings may explain why the effects of neighbor-wounding we observed did not conform to simpler expectations (Fig. 7). The emerging pattern from previous studies is that herbivore damage to an emitter plant elicits a physiological change in a receiver plant that conveys herbivore resistance and ultimately increases plant fitness in the presence of herbivores (Fig. 7a) & [3]. However, there are many additional indirect pathways by which damage to a neighbor might affect herbivory and plant fitness (Fig. 7b). For example, plant cues are information available to any organism that can access them [31] and may either directly attract or repel herbivores (Fig. 7b). In this case, the signal from a damaged “emitter” may attract herbivores under field conditions, which may in turn increase herbivory to a neighbor without any direct information exchange between the plants (Fig. 7b). This hypothesis is consistent with our observation that damage to a neighbor sometimes increased herbivory experienced by receivers in the field (Fig. 1), but it cannot account for cases in which neighbor-damage increased feeding by an herbivore in no-choice laboratory palatability trials (Fig. 4). As another possibility, the herbivore community in natural settings is often diverse, and many plant defenses are specific to particular groups of herbivores [32]. In this context, specific defenses elicited by damage to a neighbor may have no effect on some herbivores, and may even attract others. Consistent with this possibility is the fact that, in feeding assays using *Sinapis arvensis*, exposure to a damaged neighbor increased palatability to a generalist herbivore (*Spodoptera*), but decreased palatability to a specialist herbivore (*Pieris*) (Figs. 4, 5). In this situation, the net effect of damage to a neighbor will depend on the types of defensive responses and the relative abundances of alternative herbivore types in the field. Alternatively, many plants have unique responses to different herbivores based on herbivore-specific cues, and the use of mechanical damage in this study to elicit damage-induced cues may prompt a different cue than real herbivore damage.

In summary, the cues released from damaged plants have highly context-specific effects on the palatability, actual herbivore damage, phenology, and fitness of their neighbors. In a realistic multi-species field setting, we found that the consequences of receiving a signal from a damaged neighbor may be either positive (i.e. resulting in decreased palatibility) or negative (i.e. resulting in increased herbivory). Moreover, regardless of the fitness impacts, we have shown that the consequences of receiving a signal from a damaged neighbor are typically greater when the neighbor is a close relative. Overall, our study paints a more complex picture of plant information exchange than has been revealed in previous studies that find a consistent benefit of interplant cues (e.g. [5], [8], [21]). One possibility for this difference is that plant signals may operate differently in annual grasslands than in many of the systems explored previously. Past studies have most convincingly demonstrated effects of plant signals on herbivory in woody plants and vines that may coordinate their own defensive response via volatile cues [3], [8], [21], but see studies that show responses of tobacco to wounded sagebrush [7], [23] and physiological responses of Arabidopsis to volatile cues [33]. Field studies that assess the efficacy and consequences of plant signals in a variety of habitats and plant-life history types will be needed to understand the contexts in which plant signaling is a major component of plant-herbivore interactions. Accurate reporting of the effects of information transfer between plants that do not conform to our current paradigms for understanding plant signals are necessary in order to identify the contexts in which information transfer between plants is important.

## Supporting Information

Figure S1 The effect of neighbor wounding and relatedness (maternal siblings or unrelated) on plant biomass (grams, log-transformed) of three experimental plant species in the field. Least Square Mean +/− SE.
(TIF)

Figure S2 The effect of neighbor wounding and relatedness (maternal siblings or unrelated) on lifetime fruit production (log-transformed) of three experimental plant species in the field. Least Square Mean +/− SE.
(TIF)

Figure S3 The effect of neighbor wounding and relatedness (maternal siblings or unrelated) on the likelihood of feeding by generalist *Spodoptera* caterpillars in a laboratory feeding trial (as an indicator of leaf palatability to generalists). The potted plant was exposed to a damaged or undamaged neighbor in the field for 2 days. At this point the plant was moved indoors, and a feeding trial with a neonate *Spodoptera* caterpillar was initiated.
(TIF)

Table S1 Mixed model results for leaf removal on field receivers by species. When no variation was detected in a covariate for a particular species, this is noted with n/a.
(DOC)

Table S2 Mixed model results for biomass of field receivers.
(DOC)

Table S3 Mixed model results for fruit production of field receivers.
(DOC)

Table S4 Mixed model results for seed production of field receivers.
(DOC)

Table S5 Binomial model results for phenology of field receivers.
(DOC)

Table S6 Binomial model results for phenology of field receivers by species.
(DOC)

Table S7 Mixed model results for *Spodoptera* leaf removal on bioassay receivers.
(DOC)

Table S8 Mixed model results for *Spodoptera* leaf removal on bioassay receivers, by species.
(DOC)

Table S9 Binomial model results for initiation of *Spodoptera* feeding bioassay receivers.
(DOC)

Table S10 Binomial model results for initiation of Spodoptera feeding on bioassay receivers by species.
(DOC)

Table S11 Mixed model results for *Pieris* leaf removal on bioassay receivers.
(DOC)

Table S12 Mixed model results for *Pieris* caterpillar weight gain on bioassay receivers.
(DOC)
