# Supplementary figures and images for: Complex Consequences of Herbivory and Interplant Cues in Three Annual Plants
Source: PLoS One. 2012 May 31;7(5):e38105. doi: 10.1371/journal.pone.0038105 (PMC3364994; doi:10.1371/journal.pone.0038105)

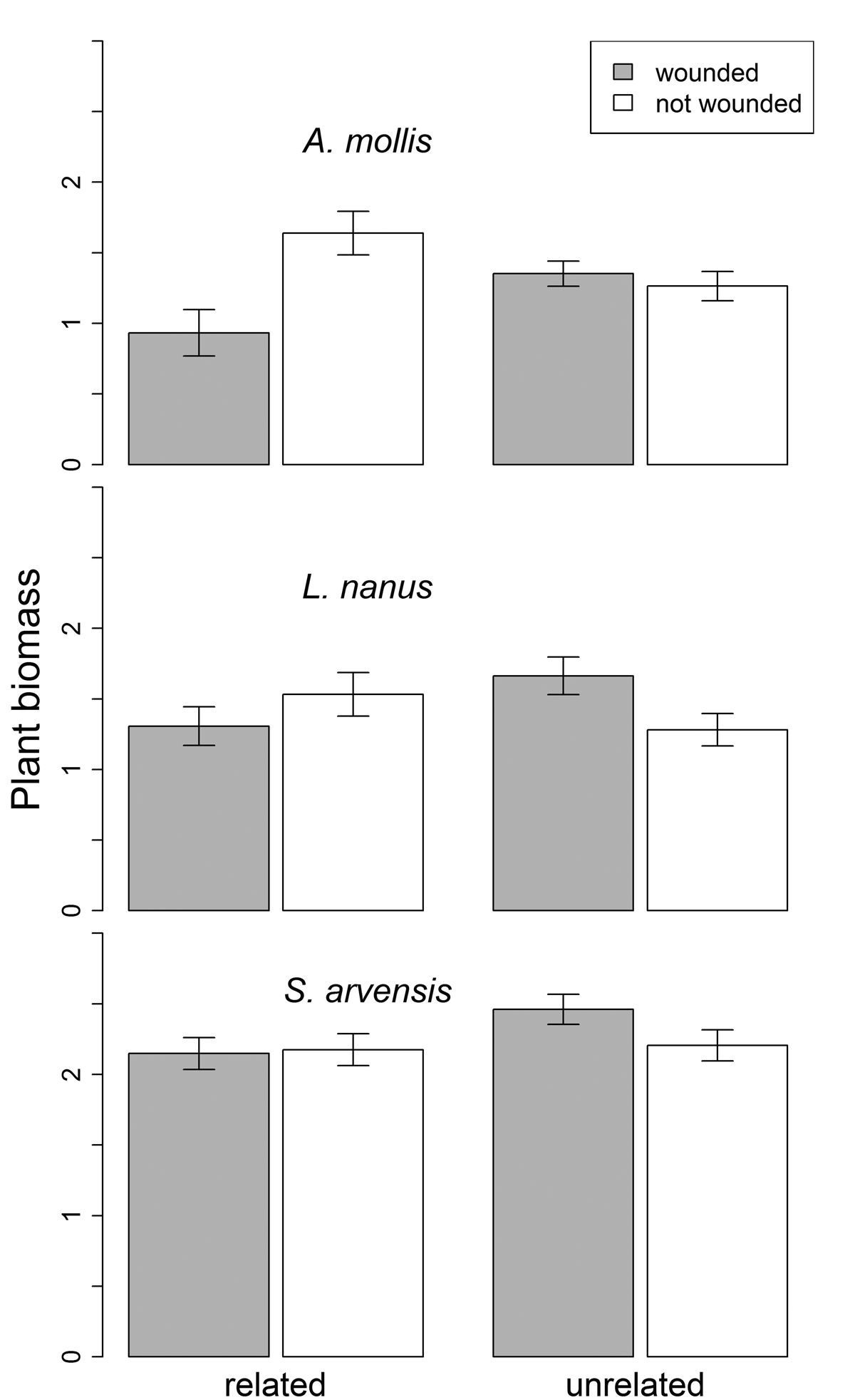

Supplement: Figure S1 — The effect of neighbor wounding and relatedness (maternal siblings or unrelated) on plant biomass (grams, log-transformed) of three experimental plant species in the field. Least Square Mean +/− SE. (TIF) [file pone.0038105.s001.tif]

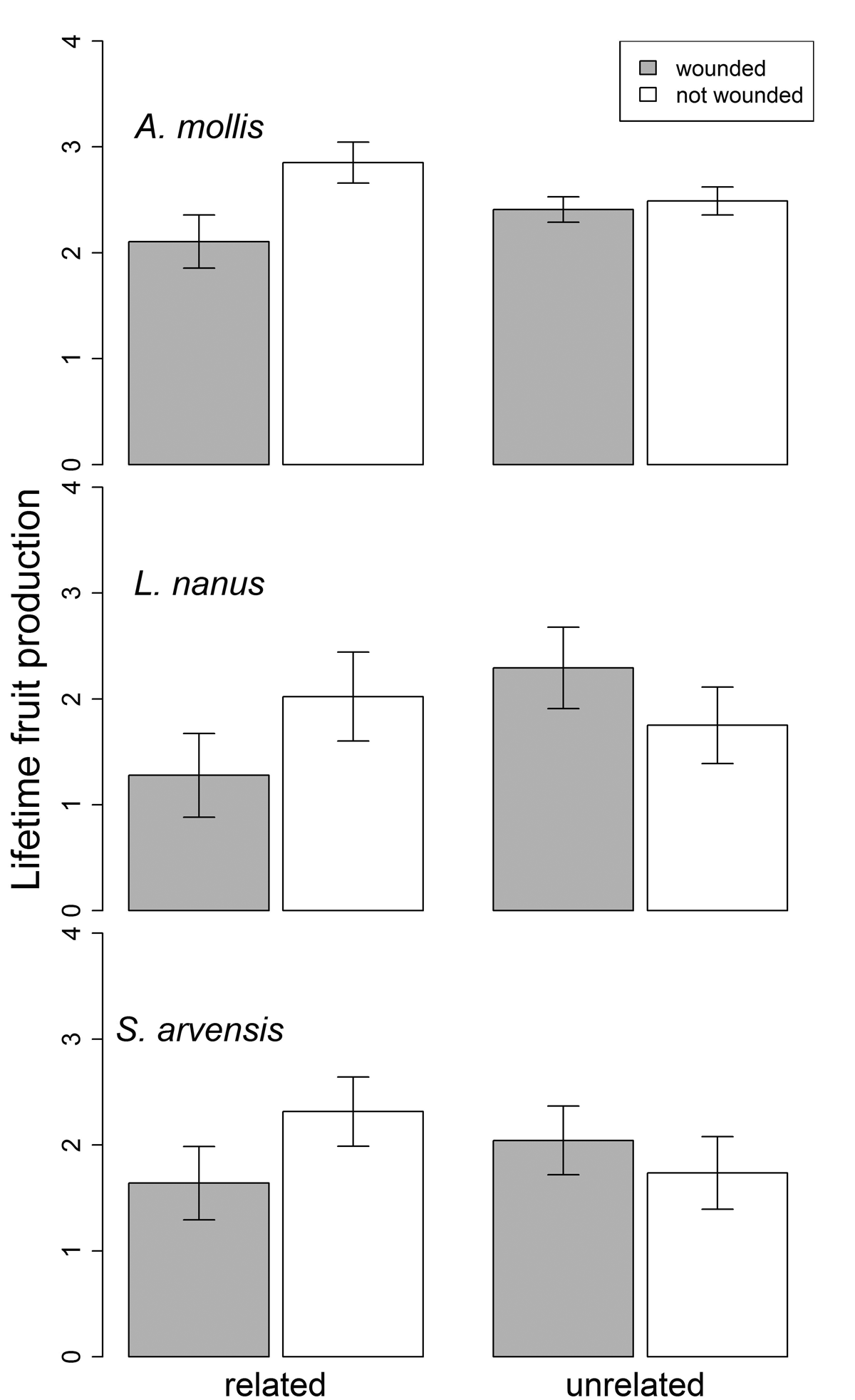

Supplement: Figure S2 — The effect of neighbor wounding and relatedness (maternal siblings or unrelated) on lifetime fruit production (log-transformed) of three experimental plant species in the field. Least Square Mean +/− SE. (TIF) [file pone.0038105.s002.tif]

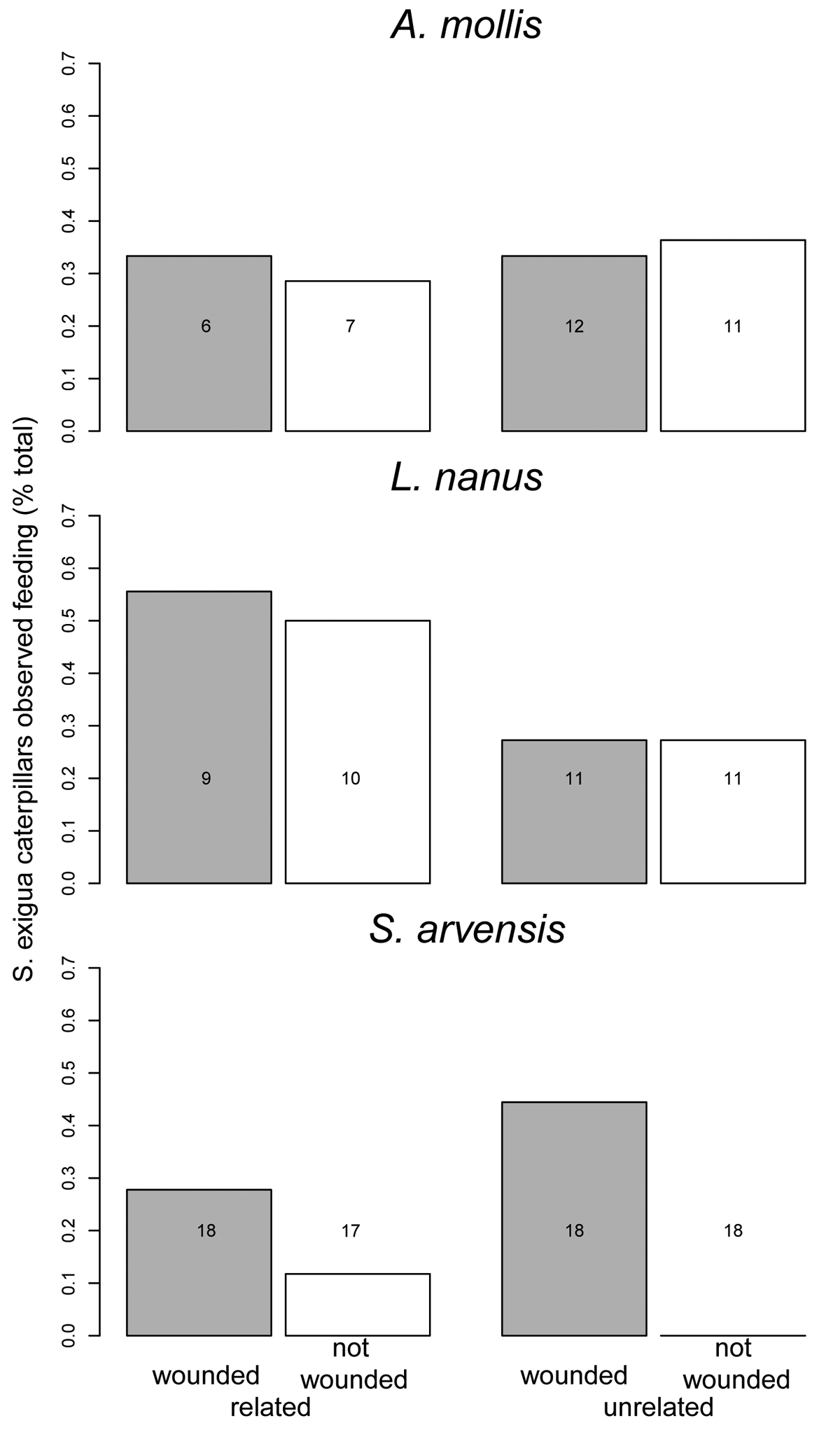

Supplement: Figure S3 — The effect of neighbor wounding and relatedness (maternal siblings or unrelated) on the likelihood of feeding by generalist Spodoptera caterpillars in a laboratory feeding trial (as an indicator of leaf palatability to generalists). The potted plant was exposed to a damaged or undamaged neighbor in the field for 2 days. At this point the plant was moved indoors, and a feeding trial with a neonate Spodoptera caterpillar was initiated. (TIF) [file pone.0038105.s003.tif]
