# Supplementary material for: Complex Consequences of Herbivory and Interplant Cues in Three Annual Plants
Source: PLoS One. 2012 May 31;7(5):e38105. doi: 10.1371/journal.pone.0038105 (PMC3364994; doi:10.1371/journal.pone.0038105)
Supplement: Table S1 — Mixed model results for leaf removal on field receivers by species. When no variation was detected in a covariate for a particular species, this is noted with n/a. (DOC) [file pone.0038105.s004.doc]

**Table S1:** Mixed model results for leaf removal on field receivers by species. When no variation was detected in a covariate for a particular species, this is noted with n/a.

| **Effect** | **num DF** | **den DF** | **F Value** | **Pr > F** | **estimate** | **std err** |
| --- | --- | --- | --- | --- | --- | --- |
| ***A. mollis*** | | | | | | |
| **date** | **2** | **38.7** | **8.91** | **0.001** |  |  |
| **wounded** | **1** | **23** | **7.66** | **0.01** |  |  |
| wounded*date | 2 | 38.7 | 1.35 | 0.27 |  |  |
| neighbor relatedness | 1 | 27.3 | 1.33 | 0.26 |  |  |
| neighbor relatedness*date | 2 | 38.7 | 0.00 | 1.00 |  |  |
| **wounded*neighbor relatedness** | **1** | **29.4** | **5.33** | **0.03** |  |  |
| wounded*neighbor relatedness*date | 2 | 38.7 | 0.99 | 0.38 |  |  |
| **pre-treatment leaf damage (receiver)** | **1** | **37.6** | **7.59** | **0.01** | 0.019 | 0.007 |
| leaf count (receiver) | 1 | 35.2 | 2.37 | 0.13 | 0.015 | 0.010 |
| leaf length (receiver) (emitter) | 1 | 35 | 1.36 | 0.25 | -0.005 | 0.004 |
| pretreatment mirid abundance (receiver) | 1 | 36.7 | 0.29 | 0.60 | 0.086 | 0.161 |
| pretreatment aphid abundance (emitter) | 1 | 37.2 | 0.00 | 0.98 | 0.004 | 0.151 |
| ***L. nanus*** | | | | | | |
| date | 2 | 32 | 1.53 | 0.23 |  |  |
| wounded | 1 | 32.4 | 0.27 | 0.60 |  |  |
| wounded*date | 2 | 32 | 2.44 | 0.10 |  |  |
| **neighbor relatedness** | **1** | **32.5** | **5.51** | **0.03** |  |  |
| neighbor relatedness*date | 2 | 32 | 0.3 | 0.75 |  |  |
| wounded*neighbor relatedness | 1 | 32.2 | 1.3 | 0.26 |  |  |
| wounded*neighbor relatedness*date | 2 | 32 | 1.26 | 0.30 |  |  |
| pre-treatment leaf damage (receiver) | 1 | 32.6 | 0.81 | 0.37 | 0.031 | 0.034 |
| **leaf count (receiver)** | **1** | **34** | **15.28** | **0.0004** | -0.010 | 0.003 |
| leaf length (receiver) (emitter) | 1 | 32.5 | 2.31 | 0.14 | 0.007 | 0.005 |
| pretreatment mirid abundance (receiver) | 1 | 32.4 | 3.06 | 0.09 | 0.785 | 0.449 |
| pretreatment aphid abundance (emitter) | 0 | n/a | n/a | n/a | n/a | n/a |
| ***S. arvernsis*** | | | | | | |
| **date** | **2** | **69.3** | **35.06** | **<.0001** |  |  |
| wounded | 1 | 58.4 | 0.05 | 0.83 |  |  |
| wounded*date | 2 | 69.3 | 1.73 | 0.18 |  |  |
| **neighbor relatedness** | **1** | **56.3** | **4.46** | **0.04** |  |  |
| neighbor relatedness*date | 2 | 69.3 | 1.05 | 0.36 |  |  |
| wounded*neighbor relatedness | 1 | 56.5 | 0.56 | 0.46 |  |  |
| **wounded*neighbor relatedness*date** | **2** | **69.3** | **3.37** | **0.04** |  |  |
| **pre-treatment leaf damage (receiver)** | **1** | **67.9** | **20.89** | **<.0001** | 0.02521 | 0.0055 |
| leaf count (receiver) | 1 | 68.3 | 0.08 | 0.77 | 0.002781 | 0.0096 |
| **leaf length (receiver) (emitter)** | **1** | **71.6** | **4.76** | **0.03** | 0.004689 | 0.0021 |
| **pretreatment mirid abundance (receiver)** | **1** | **71.5** | **7.97** | **0.006** | 0.3405 | 0.1206 |
| **pretreatment aphid abundance (emitter)** | **1** | **67** | **5.34** | **0.02** | -0.4941 | 0.2138 |
