# Supplementary material for: Complex Consequences of Herbivory and Interplant Cues in Three Annual Plants
Source: PLoS One. 2012 May 31;7(5):e38105. doi: 10.1371/journal.pone.0038105 (PMC3364994; doi:10.1371/journal.pone.0038105)
Supplement: Table S2 — Mixed model results for biomass of field receivers. (DOC) [file pone.0038105.s005.doc]

**Table S2:** Mixed model results for biomass of field receivers.

| **Effect** | | **num DF** | | **den DF** | | **F Value** | | **Pr > F** | | **estimate** | | **std err** |
| --- | --- | --- | --- | --- | --- | --- | --- | --- | --- | --- | --- | --- |
| **species** | | **2** | | **148** | | **19.07** | | **<.0001** | |  | |  |
| wounded | | 1 | | 148 | | 0.02 | | 0.8923 | |  | |  |
| species*wounded | | 2 | | 148 | | 2.48 | | 0.087 | |  | |  |
| neighbor relatedness | | 1 | | 148 | | 0.49 | | 0.4848 | |  | |  |
| species*neighbor relatedness | | 2 | | 148 | | 0.68 | | 0.5079 | |  | |  |
| **wounded*neighbor relatedness** | | **1** | | **148** | | **5.36** | | **0.022** | |  | |  |
| species*wounded*neighbor relatedness | | 2 | | 148 | | 1.28 | | 0.2823 | |  | |  |
| **pretreatment leaf damage (emitter)** | **1** | | **148** | | **16.8** | | **<.0001** | | 0.03183 | | 0.007764 | |
| **pre-treatment leaf damage (receiver)** | **1** | | **148** | | **15.96** | | **0.0001** | | -0.02411 | | 0.006034 | |
| **leaf count (receiver)** | **1** | | **148** | | **27** | | **<.0001** | | 0.0141 | | 0.002714 | |
| **leaf length (reciever)** | **1** | | **148** | | **31.4** | | **<.0001** | | 0.007875 | | 0.001405 | |
