# Supplementary material for: Complex Consequences of Herbivory and Interplant Cues in Three Annual Plants
Source: PLoS One. 2012 May 31;7(5):e38105. doi: 10.1371/journal.pone.0038105 (PMC3364994; doi:10.1371/journal.pone.0038105)
Supplement: Table S3 — Mixed model results for fruit production of field receivers. (DOC) [file pone.0038105.s006.doc]

**Table S3:** Mixed model results for fruit production of field receivers.

| **Effect** | **num DF** | **den DF** | **F Value** | **Pr > F** | **estimate** | **std err** |
| --- | --- | --- | --- | --- | --- | --- |
| **species** | **2** | **146** | **12.6** | **<.0001** |  |  |
| wounded | 1 | 146 | 0.66 | 0.4184 |  |  |
| species*wounded | 2 | 146 | 0.37 | 0.6916 |  |  |
| neighbor relatedness | 1 | 146 | 0.03 | 0.8667 |  |  |
| species*neighbor relatedness | 2 | 146 | 0.39 | 0.6747 |  |  |
| **wounded*neighbor relatedness** | **1** | **146** | **7.05** | **0.0088** |  |  |
| species*wounded*neighbor relatedness | 2 | 146 | 1.1 | 0.3371 |  |  |
| **leaf count (receiver)** | **1** | **146** | **11.25** | **0.001** | 0.01991 | 0.005933 |
| **pretreatment plant development (receiver)** | **2** | **146** | **5.09** | **0.0073** |  |  |
