# Supplementary material for: Complex Consequences of Herbivory and Interplant Cues in Three Annual Plants
Source: PLoS One. 2012 May 31;7(5):e38105. doi: 10.1371/journal.pone.0038105 (PMC3364994; doi:10.1371/journal.pone.0038105)
Supplement: Table S4 — Mixed model results for seed production of field receivers. (DOC) [file pone.0038105.s007.doc]

**Table S4:** Mixed model results for seed production of field receivers.

| **Effect** | **num DF** | **den DF** | **F Value** | **Pr > F** | **estimate** | **std err** |
| --- | --- | --- | --- | --- | --- | --- |
| **species** | **2** | **146** | **79.03** | **<.0001** |  |  |
| wounded | 1 | 146 | 2.01 | 0.1581 |  |  |
| species*wounded | 2 | 146 | 0.05 | 0.9532 |  |  |
| neighbor relatedness | 1 | 146 | 0.04 | 0.8362 |  |  |
| species*neighbor relatedness | 2 | 146 | 0.5 | 0.6103 |  |  |
| **wounded*neighbor relatedness** | **1** | **146** | **5.51** | **0.0202** |  |  |
| species*wounded*neighbor relatedness | 2 | 146 | 1.16 | 0.3154 |  |  |
| **leaf count (receiver)** | **1** | **146** | **10.74** | **0.0013** | 0.02474 | 0.007549 |
| **pretreatment plant development (receiver)** | **2** | **146** | **9.85** | **<.0001** |  |  |
