# Supplementary material for: Complex Consequences of Herbivory and Interplant Cues in Three Annual Plants
Source: PLoS One. 2012 May 31;7(5):e38105. doi: 10.1371/journal.pone.0038105 (PMC3364994; doi:10.1371/journal.pone.0038105)
Supplement: Table S5 — Binomial model results for phenology of field receivers. (DOC) [file pone.0038105.s008.doc]

**Table S5:** Binomial model results for phenology of field receivers

| **Effect** | **num DF** | **den DF** | **Chi Sq** | **Pr > chisq** |
| --- | --- | --- | --- | --- |
| wounded | 1 | 68 | 0.615 | 0.43 |
| neighbor relatedness | 1 | 68 | 1.172 | 0.28 |
| species | 1 | 68 | 0.35 | 0.55 |
| wounded*neighbor relatedness | 1 | 68 | 0.031 | 0.86 |
| **wounded*species** | **1** | **68** | **3.401** | **0.06** |
| neighbor relatedness*species | 1 | 68 | 0.105 | 0.75 |
| wounded*neighbor relatedness*species | 1 | 68 | 1.881 | 0.17 |
| **leaf length (receiver)** | **1** | **68** | **3.003** | **0.08** |
