# Supplementary material for: Complex Consequences of Herbivory and Interplant Cues in Three Annual Plants
Source: PLoS One. 2012 May 31;7(5):e38105. doi: 10.1371/journal.pone.0038105 (PMC3364994; doi:10.1371/journal.pone.0038105)
Supplement: Table S6 — Binomial model results for phenology of field receivers by species. (DOC) [file pone.0038105.s009.doc]

**Table S6:** Binomial model results for phenology of field receivers by species

| **Effect** | **num DF** | **den DF** | **Chi Sq** | **Pr > chisq** |
| --- | --- | --- | --- | --- |
| ***A. mollis*** |  |  |  |  |
| **wounded** | **1** | **35** | **4.588** | **0.03** |
| neighbor relatedness | 1 | 35 | 0.111 | 0.74 |
| wounded*neighbor relatedness | 1 | 35 | 1.222 | 0.27 |
| **leaf length (receiver)** | **1** | **35** | **8.997** | **0.003** |
| ***L. nanus*** |  |  |  |  |
| wounded | 1 | 32 | 0.864 | 0.35 |
| neighbor relatedness | 1 | 32 | 0.001 | 0.98 |
| wounded*neighbor relatedness | 1 | 32 | 0.3 | 0.58 |
| leaf length (receiver) | 1 | 32 | 2.067 | 0.15 |
