# Supplementary material for: Complex Consequences of Herbivory and Interplant Cues in Three Annual Plants
Source: PLoS One. 2012 May 31;7(5):e38105. doi: 10.1371/journal.pone.0038105 (PMC3364994; doi:10.1371/journal.pone.0038105)
Supplement: Table S7 — Mixed model results for Spodoptera leaf removal on bioassay receivers. (DOC) [file pone.0038105.s010.doc]

**Table S7:** Mixed model results for *Spodoptera* leaf removal on bioassay receivers.

| **Effect** | **num DF** | **den DF** | **F Value** | **Pr > F** | **estimate** | **std err** |
| --- | --- | --- | --- | --- | --- | --- |
| species | 2 | 51.9 | 1.83 | 0.1711 |  |  |
| wounded | 1 | 120 | 0.25 | 0.6154 |  |  |
| **species*wounded** | **2** | **117** | **2.65** | **0.0747** |  |  |
| neighbor relatedness | 1 | 130 | 0.36 | 0.5503 |  |  |
| species*neighbor relatedness | 2 | 128 | 0.01 | 0.9876 |  |  |
| wounded*neighbor relatedness | 1 | 122 | 0.91 | 0.3431 |  |  |
| species*wounded*neighbor relatedness | 2 | 119 | 0.61 | 0.5465 |  |  |
| **pretreatment leaf damage (bioassay receiver)** | **1** | **129** | **4.77** | **0.0308** | -0.09086 | 0.04161 |
| **pretreatment leaf spotting (emitter)** | **1** | **132** | **15.74** | **0.0001** | 0.1339 | 0.03376 |
