# Supplementary material for: Complex Consequences of Herbivory and Interplant Cues in Three Annual Plants
Source: PLoS One. 2012 May 31;7(5):e38105. doi: 10.1371/journal.pone.0038105 (PMC3364994; doi:10.1371/journal.pone.0038105)
Supplement: Table S8 — Mixed model results for Spodoptera leaf removal on bioassay receivers, by species. (DOC) [file pone.0038105.s011.doc]

**Table S8:** Mixed model results for *Spodoptera* leaf removal on bioassay receivers, by species.

| **Effect** | **num DF** | **den DF** | **F Value** | **Pr > F** | **estimate** | **std err** |
| --- | --- | --- | --- | --- | --- | --- |
| ***A. Mollis*** |  |  |  |  |  |  |
| wounded | 1 | 31.5 | 0.98 | 0.329 |  |  |
| neighbor relatedness | 1 | 32.5 | 0.58 | 0.451 |  |  |
| wounded*neighbor relatedness | 1 | 29 | 0.36 | 0.556 |  |  |
| **pretreatment leaf damage (bioassay receiver)** | **1** | **37.6** | **4.29** | **0.045** | -0.07941 | 0.0384 |
| **pretreatment leaf spotting (emitter)** | **1** | **32.6** | **7.34** | **0.011** | 0.1003 | 0.037 |
| ***L. Nanus*** |  |  |  |  |  |  |
| wounded | 1 | 29.5 | 1.66 | 0.208 |  |  |
| neighbor relatedness | 1 | 32.6 | 0.54 | 0.467 |  |  |
| wounded*neighbor relatedness | 1 | 32.5 | 1.78 | 0.191 |  |  |
| pretreatment leaf damage (bioassay receiver) | 1 | 33.1 | 0.56 | 0.461 | 0.1174 | 0.1575 |
| pretreatment leaf spotting (emitter) | 1 | 29.4 | 0.25 | 0.624 | 0.05115 | 0.1032 |
| ***S. Arvensis*** |  |  |  |  |  |  |
| **wounded** | **1** | **58.3** | **9.02** | **0.0039** |  |  |
| neighbor relatedness | 1 | 58.3 | 0.63 | 0.4323 |  |  |
| wounded*neighbor relatedness | 1 | 58.7 | 0.52 | 0.4738 |  |  |
| pretreatment leaf damage (bioassay receiver) | 1 | 65.8 | 1.49 | 0.227 | -0.795 | 0.6519 |
| **pretreatment leaf spotting (emitter)** | **1** | **64.7** | **14.77** | **0.0003** | 0.2035 | 0.053 |
