# Supplementary material for: Complex Consequences of Herbivory and Interplant Cues in Three Annual Plants
Source: PLoS One. 2012 May 31;7(5):e38105. doi: 10.1371/journal.pone.0038105 (PMC3364994; doi:10.1371/journal.pone.0038105)
Supplement: Table S9 — Binomial model results for initiation of Spodoptera feeding bioassay receivers. (DOC) [file pone.0038105.s012.doc]

**Table S9:** Binomial model results for initiation of *Spodoptera* feeding bioassay receivers

| **Effect** | **num DF** | **den DF** | **Chi Sq** | **Pr > chisq** |
| --- | --- | --- | --- | --- |
| **wounded** | **1** | **136** | **4.463** | **0.04** |
| neighbor relatedness | 1 | 136 | 0.467 | 0.44 |
| **species** | **2** | **136** | **4.877** | **0.09** |
| wounded*neighbor relatedness | 1 | 136 | 0.492 | 0.48 |
| **wounded*species** | **2** | 136 | **6.561** | **0.04** |
| neighbor relatedness*species | 2 | 136 | 2.091 | 0.35 |
| wounded*neighbor relatedness*species | 2 | 136 | 3.615 | 0.16 |
