# Supplementary material for: Complex Consequences of Herbivory and Interplant Cues in Three Annual Plants
Source: PLoS One. 2012 May 31;7(5):e38105. doi: 10.1371/journal.pone.0038105 (PMC3364994; doi:10.1371/journal.pone.0038105)
Supplement: Table S10 — Binomial model results for initiation of Spodoptera feeding on bioassay receivers by species. (DOC) [file pone.0038105.s013.doc]

**Table S10:** Binomial model results for initiation of Spodoptera feeding on bioassay receivers by species

| **Effect** | **num DF** | **den DF** | **Chi Sq** | **Pr > chisq** |
| --- | --- | --- | --- | --- |
| ***A. mollis*** |  |  |  |  |
| wounded | 1 | 32 | 0.001 | 0.99 |
| neighbor relatedness | 1 | 32 | 0.061 | 0.81 |
| wounded*neighbor relatedness | 1 | 32 | 0.057 | 0.81 |
| ***L. nanus*** |  |  |  |  |
| wounded | 1 | 37 | 0.03 | 0.86 |
| **neighbor relatedness** | **1** | **37** | **2.792** | **0.09** |
| wounded*neighbor relatedness | 1 | 37 | 0.028 | 0.87 |
| ***S. arvensis*** |  |  |  |  |
| **wounded** | **1** | **67** | **10.83** | **<0.001** |
| neighbor relatedness | 1 | 67 | 0.087 | 0.77 |
| **wounded*neighbor relatedness** | **1** | **67** | **4.021** | **0.04** |
