# Supplementary material for: Complex Consequences of Herbivory and Interplant Cues in Three Annual Plants
Source: PLoS One. 2012 May 31;7(5):e38105. doi: 10.1371/journal.pone.0038105 (PMC3364994; doi:10.1371/journal.pone.0038105)
Supplement: Table S11 — Mixed model results for Pieris leaf removal on bioassay receivers. (DOC) [file pone.0038105.s014.doc]

**Table S11:** Mixed model results for *Pieris* leaf removal on bioassay receivers.

| **Effect** | **num DF** | **den DF** | **F Value** | **Pr > F** | **estimate** | **std err** |
| --- | --- | --- | --- | --- | --- | --- |
| **wounded** | **1** | **50.7** | **3.86** | **0.612** |  |  |
| **neighbor relatedness_C** | **1** | **54.1** | **6.86** | **0.145** |  |  |
| **wounded*neighbor relatedness_C** | **1** | **51.7** | **13.7** | **0.006** |  |  |
| **pretreatment leaf spotting (emitter)** | **1** | **52.8** | **8.15** | **0.007** | 0.1413 | 0.04951 |
| **pretreatment mirid abundance (emitter)** | **1** | **55.8** | **4.23** | **0.011** | 0.2507 | 0.1219 |
| **Leaf count (bioassay receiver)** | **1** | **58** | **4.59** | **0.036** |  |  |
| **Pretreatment plant stage (bioassay receiver)** | **2** | **46.9** | **9.34** | **0.001** |  |  |
| **pretreatment leaf spotting (bioassay receiver)** | **1** | **56.6** | **6.62** | **0.014** | 0.05042 | 0.01959 |
| **pretreatment aphid abundance (bioassay receiver)** | **1** | **56.2** | **7.68** | **0.046** | 0.9339 | 0.3369 |
