# Supplementary material for: Complex Consequences of Herbivory and Interplant Cues in Three Annual Plants
Source: PLoS One. 2012 May 31;7(5):e38105. doi: 10.1371/journal.pone.0038105 (PMC3364994; doi:10.1371/journal.pone.0038105)
Supplement: Table S12 — Mixed model results for Pieris caterpillar weight gain on bioassay receivers. (DOC) [file pone.0038105.s015.doc]

**Table S12:** Mixed model results for *Pieris* caterpillar weight gain on bioassay receivers.

| **Effect** | **num DF** | **den DF** | **F Value** | **Pr > F** | **estimate** | **std err** |
| --- | --- | --- | --- | --- | --- | --- |
| wounded | 1 | 33.7 | 1.18 | **0.3321** |  |  |
| **neighbor relatedness_C** | **1** | **33.3** | **9.61** | **0.0292** |  |  |
| wounded*neighbor relatedness_C | 1 | 33.7 | 0.55 | 0.2729 |  |  |
| **leaf length (bioassay receiver)** | **1** | **41.6** | **6.23** | **0.0404** | 0.005972 | 0.002392 |
| **pretreatment plant stage (bioassay reciever)** | **2** | **40.6** | **3.24** | **0.3742** |  |  |
